# Supplementary material for: Paradise fish (Macropodus opercularis) as a complementary translational model for emotional and cognitive function
Source: Commun Biol. 2025 Jul 29;8:1125. doi: 10.1038/s42003-025-08556-0 (PMC12307770; doi:10.1038/s42003-025-08556-0)
Supplement: Supplementary file 2 — Supplementary Materials [file 42003_2025_8556_MOESM2_ESM.pdf]

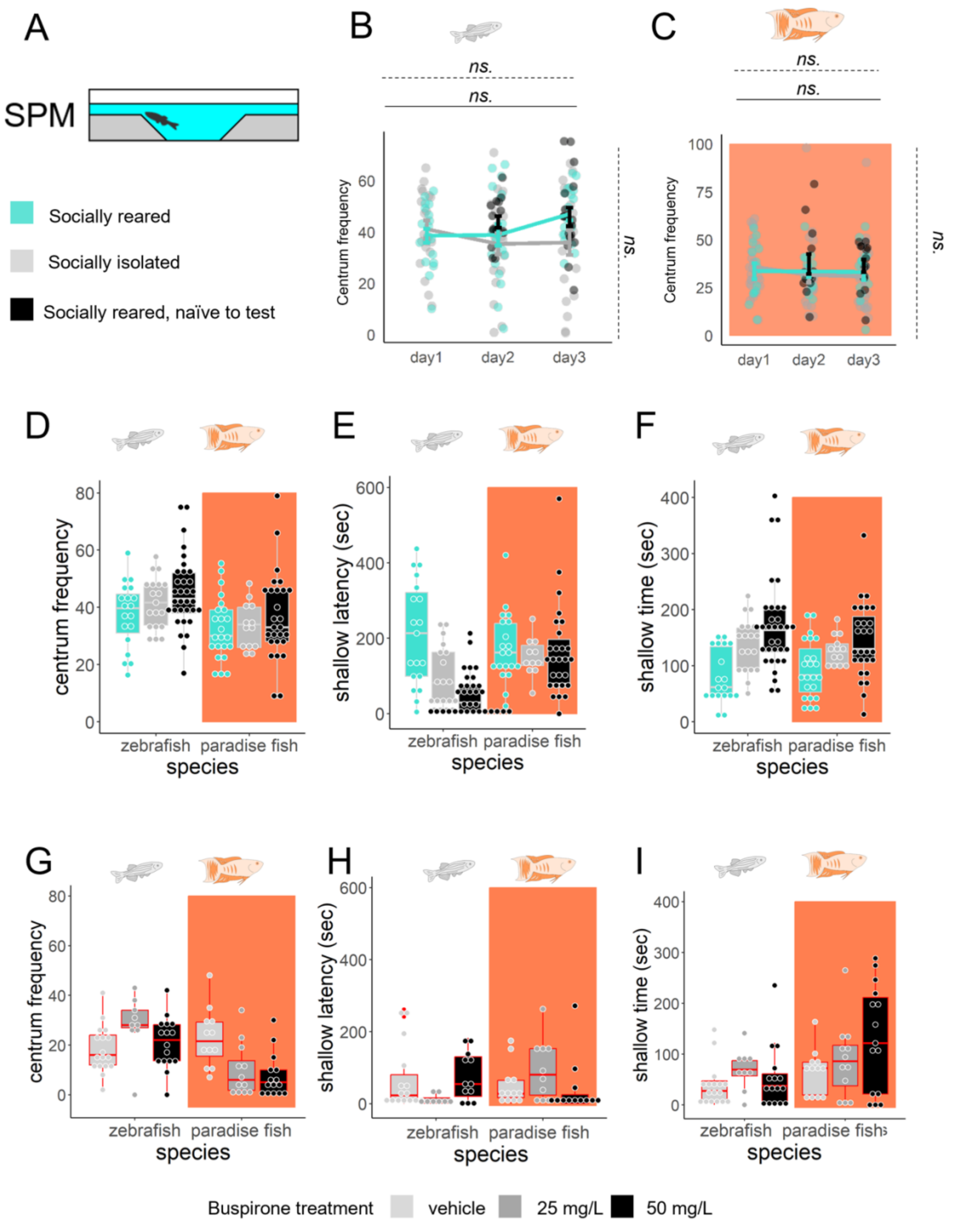

Supplementary Figure 1. Exploration variables as markers of anxiety. A) Zebrafish and paradise fish were tested in the SPM test following social isolation stress. Centrum enter frequencies at each testing day in zebrafish and (B) and paradise fish (C). Horizontal solid or dashed lines with asterisk represent significant main effect of testing day or significant

interaction between testing days and groups, respectively. Vertical dashed lines with asterisk represent significant main effect of the groups. D-I) Centrum enter frequencies, shallow arm latencies and time spent in shallow arms following social isolation (D, E, F) or buspirone treatment (G, H, I).

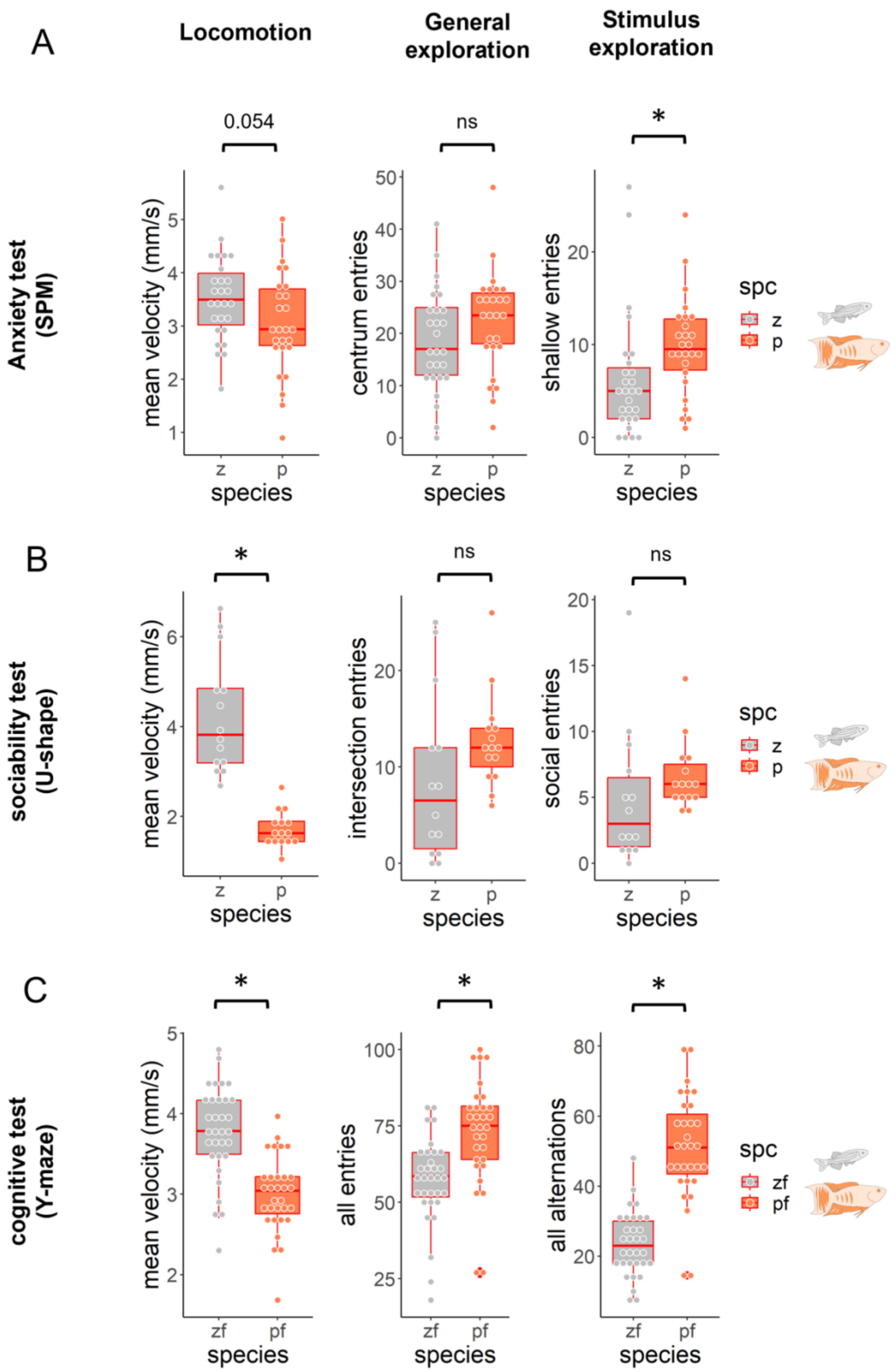

Supplementary Figure 2. Indicators of locomotion (left column), general exploration (middle column) or stimulus exploration (right column) in an anxiety-(A), a sociability-(B) and a cognitive test (C). * Indicate significant difference between the two species.

Supplementary Tables

Supplementary table 1. Statistical Results from Experiment 1

**Species**	**Condition**	**variable**	**test statistics**			**n**
**Swimming velocity across contexts**						
ANOVA main effect (~challenge type)			*F*		p-value	
zebra	-	velocity (mm/s)	0.37	-	0.693	
paradise	-	velocity (mm/s)	4.02	-	0.025	
**Swimming velocity comparisons**						
posthoc contrasts (to intra-species challenge)			*Est.*	*SE*	p-value	
paradise	Inter-species challenge	velocity (mm/s)	0.45	0.21	0.035	
paradise	Double-species challenge	velocity (mm/s)	0.56	0.21	0.009	
**Species comparison of swimming speed**						
*ANOVA main effect (~species*challenge type)*			*F*		p-value	
paradise & zebra	-	velocity (mm/s)	110.32	-	<0.0001	
**Intersection zone visits**						
*ANOVA main effect (~species*challenge type)*			*F*		p-value	
paradise & zebra	-	velocity (mm/s)	9.31	-	0.003	
**Zone preference comparisons (Zebrafish)**						intra/**inter**/double: 15,**17**,16 (paradise), 14,**14**,15 (zebra)
posthoc contrasts			*Est.*	*SE*	p-value	
Intra-species challenge						
zebra	non-social vs. intersection	time (s)	26.11	9.83	0.024	
	non-social vs. social		-36.17	9.83	0.001	
	intersection vs. social		-62.28	9.83	<0.0001	
Inter-species challenge						
zebra	non-social vs. intersection	time (s)	39.51	9.83	0.000	
	non-social vs. social		-6.25	9.83	0.801	
	intersection vs. social		-45.76	9.83	<0.0001	
Double-species challenge						
zebra	non-social vs. intersection	time (s)	33.63	9.49	0.002	
	non-social vs. social		-22.31	9.49	0.053	
	intersection vs. social		-55.94	9.49	<0.0001	
Intra-species challenge						

Supplementary table 1. Statistical Results from Experiment 1

Species	Condition	variable	test statistics			n
zebra	non-social vs. intersection	entires	-4.21	2.31	0.165	
	non-social vs. social		-0.43	2.31	0.981	
	intersection vs. social		3.79	2.31	0.232	
Inter-species challenge						
zebra	non-social vs. intersection	entires	-4.21	2.31	0.165	
	non-social vs. social		0.36	2.31	0.986	
	intersection vs. social		4.57	2.31	0.121	
Double-species challenge						
zebra	non-social vs. intersection	entires	-4.20	2.23	0.147	
	non-social vs. social		-1.33	2.23	0.821	
	intersection vs. social		2.87	2.23	0.405	
Intra-species challenge			*Est.*	*SE*	p-value	
paradise	non-social vs. intersection	time (s)	36.55	5.62	<.0001	
	non-social vs. social		8.23	5.62	0.311	
	intersection vs. social		-28.32	5.62	<.0001	
Inter-species challenge						
paradise	non-social vs. intersection	time (s)	47.59	5.28	<.0001	
	non-social vs. social		22.16	5.28	<.0001	
	intersection vs. social		-25.42	5.28	<.0001	
Double-species challenge						
paradise	non-social vs. intersection	time (s)	44.81	5.44	<.0001	
	non-social vs. social		13.85	5.44	0.032	
	intersection vs. social		-30.96	5.44	<.0001	
Intra-species challenge						
paradise	non-social vs. intersection	entires	-5.47	1.64	0.003	
	non-social vs. social		0.60	1.64	0.929	
	intersection vs. social		6.07	1.64	0.001	
Inter-species challenge						
paradise	non-social vs. intersection	entires	-5.53	1.54	0.001	
	non-social vs. social		1.88	1.54	0.443	

Supplementary table 1. Statistical Results from Experiment 1

Species	Condition	variable	test statistics			n
	intersection vs. social		7.41	1.54	<.0001	
Double-species challenge						
paradise	non-social vs. intersection	entires	-6.00	1.59	0.001	
	non-social vs. social		0.50	1.59	0.947	
	intersection vs. social		6.50	1.59	0.000	

Supplementary table 2. Statistical Results from Experiment 2

Species	Condition	variable	test statistics			n
**slalom test**						
ANOVA main effect (~group size)			*F*		p-value	
paradise & zebra	30 dpf	mean exp. lat.	4.71	-	0.010	
posthoc contrasts			*Est.*	*SE*	p-value	
group of 1						
paradise & zebra	8 dpf zebra vs 30 dpf zebra	mean exp. lat.	0.00	0.06	0.986	
	8 dpf zebra vs 30 dpf paradise		-0.11	0.07	0.246	
	30 dpf zebra vs 30 dpf paradise		-0.11	0.08	0.246	
group of 2						
paradise & zebra	8 dpf zebra vs 30 dpf zebra	mean exp. lat.	0.15	0.07	0.035	
	8 dpf zebra vs 30 dpf paradise		-0.07	0.07	0.267	
	30 dpf zebra vs 30 dpf paradise		-0.23	0.08	0.009	group of 1 / **group of 2**: 21,**19** (30 dpf paradise), 24,**30** (8 dpf zebra), 26,**30** (30 dpf zebra)
Fisher Exact Test						
paradise & zebra	All conditions	exploration success	-	-	0.001	
Gaussian mixture model			*number of peaks*	*BIC*	*BIC normalised*	
zebra	8 dpf, group of 1	mean exp. lat.	1.00	-11.89	-0.344	
			2.00	-6.64	1.127	
			3.00	-13.45	-0.782	
	8 dpf, group of 2		1.00	-11.15	0.641	
			2.00	-11.48	0.511	
			3.00	-15.68	-1.152	
	30 dpf, group of 1		1.00	-1.79	0.949	
			2.00	-5.18	0.095	
			3.00	-9.71	-1.044	
	30 dpf, group of 2		1.00	-12.53	-0.388	
			2.00	-7.46	1.136	
			3.00	-13.73	-0.747	

Supplementary table 2. Statistical Results from Experiment 2

Species	Condition	variable	test statistics		n
paradise	30 dpf, group of 1	1.00	-10.75	0.909	
		2.00	-14.43	0.163	
		3.00	-20.52	-1.071	
	30 dpf, group of 2	1.00	-2.60	0.991	
		2.00	-7.32	0.018	
		3.00	-12.30	-1.009	

Supplementary table 3. Statistical Results from Experiment 3

Species	Condition	variable	test statistics			n
**showjump test**						
*ANOVA main effect (~rearing*testing day)*			*F*		p-value	
zebra	group	mean latency	5.00		0.003	
	day		0.74		0.479	
	group*day		0.08		0.920	
posthoc contrasts			*Est.*	*SE*	p-value	
zebra	iso control vs soc control	mean latency	0.17	0.07	0.017	
	iso control vs soc naive		0.27	0.08	0.001	
	soc control vs soc naive		0.11	0.08	0.166	
*ANOVA main effect (~rearing*testing day)*			*F*		p-value	
zebra	group	all entires	4.38		0.006	
	day		1.01		0.367	
	group*day		1.72		0.183	
posthoc contrasts			*Est.*	*SE*	p-value	
zebra	iso control vs soc control	all entires	-12.00	6.51	0.101	
	iso control vs soc naive		-24.00	7.61	0.006	
	soc control vs soc naive		-12.00	7.61	0.116	
**swimming plus-maze test**						
*ANOVA main effect (~rearing*testing day)*			*F*		p-value	
zebra	group	shallow latency	8.32		0.000	
	day		1.13		0.327	
	group*day		3.88		0.023	
posthoc contrasts			*Est.*	*SE*	p-value	socially reared/**isolated**/test naive d2/*test naive day3*: 13,**23**,13,*14* (paradise fish), 19,**19**,15,*18* (zebrafish)
zebra	day 1	shallow latency				
	iso control vs soc control		2.37	52.20	1.000	
	day 2					
	iso control vs soc control		141.24	52.20	0.023	
	iso control vs soc naive		193.92	55.60	0.004	
	soc control vs soc naive		52.68	55.60	0.691	
	day 3					
	iso control vs soc control		203.37	52.20	0.001	
	iso control vs soc naive		209.59	53.00	0.001	
	soc control vs soc naive		6.22	53.00	1.000	
*ANOVA main effect (~rearing*testing day)*			*F*		p-value	
zebra	group	shallow entries	9.10		<0.0001	
	day		0.08		0.920	
	group*day		2.59		0.078	
posthoc contrasts			*Est.*	*SE*	p-value	
zebra	day 1	shallow entries				
	iso control vs soc control		-0.21	2.74	1.000	
	day 2					
	iso control vs soc control		-5.58	2.74	0.087	
	iso control vs soc naive		-11.87	2.91	0.001	
	soc control vs soc naive		-6.29	2.91	0.087	

Supplementary table 3. Statistical Results from Experiment 3

Species	Condition	variable	test statistics			n
	day 3					
	iso control vs soc control		-8.95	2.74	0.006	
	iso control vs soc naive		-8.74	2.77	0.006	
	soc control vs soc naive		0.21	2.77	1.000	
**showjump test**						
*ANOVA main effect (~rearing*testing day)*			*F*		p-value	
paradise	group	mean latency	0.83		0.479	
	day		2.14		0.122	
	group*day		0.82		0.444	
*ANOVA main effect (~rearing*testing day)*			*F*		p-value	
paradise	group	all entires	1.75		0.159	
	day		6.26		0.003	
	group*day		2.28		0.106	
posthoc contrasts			*Est.*	*SE*	p-value	
paradise	day 1 vs 2	all entires	11.11	8.48	1.311	
	day 1 vs 3		11.34	8.48	1.337	
	day 2 vs 3		0.23	8.57	0.027	
**swimming plus-maze test**						
*ANOVA main effect (~rearing*testing day)*			*F*		p-value	
paradise	group	shallow latency	0.49		0.688	
	day		0.09		0.917	
	group*day		0.10		0.901	
*ANOVA main effect (~rearing*testing day)*			*F*		p-value	
paradise	group	shallow entries	3.68		0.028	
	day		2.02		0.137	
	group*day		0.34		0.795	

Supplementary table 4. Statistical Results from Experiment 4

Species	Condition	variable	test statistics			n
**swimming plus-maze (social isolation)**						
ANOVA main effect (~group)			*F*		p-value	
paradise & zebra		anxiety score	14.32		<0.0001	
posthoc contrasts			*Est.*	*SE*	p-value	
zebra		anxiety score	-1.25	0.36	0.001	
paradise		anxiety score	-0.72	0.39	0.068	
**swimming plus-maze (buspirone treatment)**						
Kruskal-Wallis main effect (~group)			*chi-squared*		p-value	
zebra		anxiety score	5.20		0.074	
posthoc contrasts						
	veh vs 25 mg	anxiety score			0.048	
	veh vs 50 mg	anxiety score			0.792	
	25 vs 50 mg	anxiety score			0.202	
Kruskal-Wallis main effect (~group)			*chi-squared*		p-value	
paradise		anxiety score	9.25		0.010	veh/**25**/50 mg/L:12,**12**,15 (zebrafish), 17,**9**,16 (paradise fish)
posthoc contrasts						
	veh vs 25 mg	anxiety score			0.582	
	veh vs 50 mg	anxiety score			0.014	
	25 vs 50 mg	anxiety score			0.025	
ANOVA main effect (~group)			*F*		p-value	
zebra		mean velocity	5.99		0.006	
posthoc contrasts			*Est.*	*SE*	p-value	
	veh vs 25 mg	mean velocity	-1.20	0.41	0.009	
	veh vs 50 mg	mean velocity	0.10	0.35	0.784	
	25 vs 50 mg	mean velocity	1.29	0.41	0.009	
ANOVA main effect (~group)			*F*		p-value	
paradise		mean velocity	21.23		<0.0001	
posthoc contrasts			*Est.*	*SE*	p-value	
	veh vs 25 mg	mean velocity	1.58	0.31	<.0001	
	veh vs 50 mg	mean velocity	1.80	0.31	<.0001	
	25 vs 50 mg	mean velocity	0.21	0.30	0.488	

Supplementary table 5. Statistical Results from Experiment 5

Species	Condition	variable	repeatability	lower CI	signiciant	n
**Swimming plus-maze and open-tank repeatability**						
paradise	OT velocity		0.64	0.36	*	
paradise	OT immobility		0.67	0.41	*	
paradise	OT avoidance		0.00	0.00	ns.	
paradise	SPM avoidance		0.19	0.00	ns.	
paradise	SPM velocity		0.74	0.50	*	
paradise	SPM immobility		0.81	0.64	*	zebrafish/**paradise fish**: 24, **24**
zebra	OT velocity		0.71	0.50	*	
zebra	OT immobility		0.67	0.45	*	
zebra	OT avoidance		0.64	0.39	*	
zebra	SPM avoidance		0.03	0.00	ns.	
zebra	SPM velocity		0.23	0.00	ns.	
zebra	SPM immobility		0.18	0.00	ns.	

Supplementary table 6. Statistical Results from Experiment 6

Species	Condition	variable	test statistics			n
**Y-maze**						
*ANOVA main effect (~action type*species)*			*F*		p-value	
paradise & zebra	action type	count	302.91		<0.0001	
	species		15.22		<0.0001	
	action type*species		82.67		<0.0001	
posthoc contrasts (zebra vs paradise fish)			*Est.*	*SE*	p-value	
paradise & zebra	indirect revisits	count	11.29	2.25	<.0001	
	direct revisits		0.25	2.25	0.913	zebrafish/**paradise fish**: 31, **32**
	alternations		-27.43	2.25	<.0001	
t.test (zebra vs paradise fish)						
paradise & zebra		alternation %	-11.594		<0.0001	
		mean velocity	5.9038		<0.0001	

Supplementary table 7. Direct comparisons of locomotive and exploratiove variables

Species	Condition	variable	test statistics		n
**Sociability**					
t.test (zebra vs paradise fish)			*t*	p-value	
paradise & zebra		mean velocity	-6.95	<0.0001	zebrafish/**paradise fish**: 15, **14**
		intersection entires	1.52	0.143	
		social entries	1.14	0.268	
**Swimming plus-maze**					
t.test (zebra vs paradise fish)					
paradise & zebra		mean velocity	-1.97	0.054	zebrafish/**paradise fish**: 13, **19**
		centrum entires	1.37	0.178	
		shallow arm entries	2.14	0.038	
**y-maze**					
t.test (zebra vs paradise fish)					
paradise & zebra		mean velocity	-5.90	<0.0001	zebrafish/**paradise fish**: 31, **32**
		all entries	3.89	<0.0001	
		all alternations	8.60	<0.0001	
