# Supplementary material for: Paradise fish (Macropodus opercularis) as a complementary translational model for emotional and cognitive function
Source: Commun Biol. 2025 Jul 29;8:1125. doi: 10.1038/s42003-025-08556-0 (PMC12307770; doi:10.1038/s42003-025-08556-0)
Supplement: Supplementary file 3 — Reporting Summary [file 42003_2025_8556_MOESM3_ESM.pdf]

nature portfolio

Corresponding author(s): Máté Varga (COMMSBIO-24-6436)

Last updated by author(s): Jul 3, 2025

Reporting Summary

Nature Portfolio wishes to improve the reproducibility of the work that we publish. This form provides structure for consistency and transparency in reporting. For further information on Nature Portfolio policies, see our Editorial Policies and the Editorial Policy Checklist.

Statistics

For all statistical analyses, confirm that the following items are present in the figure legend, table legend, main text, or Methods section.

n/a	Confirmed	
☐	☒	The exact sample size (*n*) for each experimental group/condition, given as a discrete number and unit of measurement
☐	☒	A statement on whether measurements were taken from distinct samples or whether the same sample was measured repeatedly
☐	☒	The statistical test(s) used AND whether they are one- or two-sided<br>*Only common tests should be described solely by name; describe more complex techniques in the Methods section.*
☐	☒	A description of all covariates tested
☐	☒	A description of any assumptions or corrections, such as tests of normality and adjustment for multiple comparisons
☐	☒	A full description of the statistical parameters including central tendency (e.g. means) or other basic estimates (e.g. regression coefficient) AND variation (e.g. standard deviation) or associated estimates of uncertainty (e.g. confidence intervals)
☐	☒	For null hypothesis testing, the test statistic (e.g. F, t, r) with confidence intervals, effect sizes, degrees of freedom and P value noted<br>*Give P values as exact values whenever suitable.*
☒	☐	For Bayesian analysis, information on the choice of priors and Markov chain Monte Carlo settings
☐	☒	For hierarchical and complex designs, identification of the appropriate level for tests and full reporting of outcomes
☐	☒	Estimates of effect sizes (e.g. Cohen's *d*, Pearson's *r*), indicating how they were calculated

Our web collection on statistics for biologists contains articles on many of the points above.

Software and code

Policy information about availability of computer code

Data collection	Behavioural data were collected using the commercially available Noldus Ethovision XT15 software.
Data analysis	Data were analyzed using custom-made codes in the R statistical environment. Version number and packages are cited in the main text. No unpublished functions were used in the study.

For manuscripts utilizing custom algorithms or software that are central to the research but not yet described in published literature, software must be made available to editors and reviewers. We strongly encourage code deposition in a community repository (e.g. GitHub). See the Nature Portfolio guidelines for submitting code & software for further information.

Data

Policy information about availability of data

All manuscripts must include a data availability statement. This statement should provide the following information, where applicable:

- Accession codes, unique identifiers, or web links for publicly available datasets
- A description of any restrictions on data availability
- For clinical datasets or third party data, please ensure that the statement adheres to our policy

All data of the study is available at the data repository ZENODO under the title "Paradise fish (Macropodus opercularis) as novel translational model for emotional and cognitive function - RAW DATA". Doi: 10.5281/zenodo.14028710
; URL: DOI https://doi.org/10.5281/zenodo.14028710; license: Creative Commons Attribution Share Alike 4.0 International

Human research participants

Policy information about studies involving human research participants and Sex and Gender in Research.

Reporting on sex and gender	NA
Population characteristics	NA
Recruitment	NA
Ethics oversight	NA

Note that full information on the approval of the study protocol must also be provided in the manuscript.

Field-specific reporting

Please select the one below that is the best fit for your research. If you are not sure, read the appropriate sections before making your selection.

☒ Life sciences ☐ Behavioural & social sciences ☐ Ecological, evolutionary & environmental sciences

For a reference copy of the document with all sections, see nature.com/documents/nr-reporting-summary-flat.pdf

Life sciences study design

All studies must disclose on these points even when the disclosure is negative.

Sample size	Sample sizes were determined according to previous studies and pilot experiments. Sample sizes were chosen to be appropriate for revealing a 20% effect size which we consider biologically relevant in the behavioural assays of the study.
Data exclusions	No data were excluded during the analysis
Replication	Similar results were found in multiple experiments and several cohorts of animals were used in the study.
Randomization	Exposure to pharmacological agents and behavioural testing were done in a previously fully randomized manner
Blinding	Totally blind experimentation is not possible in high-throughput behavioural assays but due to the throughput (12-24 animals in a single trial) and apriori randomization there is no space for any experimenter-driven bias.

Reporting for specific materials, systems and methods

We require information from authors about some types of materials, experimental systems and methods used in many studies. Here, indicate whether each material, system or method listed is relevant to your study. If you are not sure if a list item applies to your research, read the appropriate section before selecting a response.

Materials & experimental systems

n/a	Involved in the study	
☒	☐	Antibodies
☒	☐	Eukaryotic cell lines
☒	☐	Palaeontology and archaeology
☐	☒	Animals and other organisms
☒	☐	Clinical data
☒	☐	Dual use research of concern

Methods

n/a	Involved in the study	
☒	☐	ChIP-seq
☒	☐	Flow cytometry
☒	☐	MRI-based neuroimaging

Animals and other research organisms

Policy information about studies involving animals; ARRIVE guidelines recommended for reporting animal research, and Sex and Gender in Research

Laboratory animals	Wild type zebrafish of the AB strain between 8 and 30 days post fertilisation were used. Wild type paradise fish (no specific lab strain idenfication) at 30 days post fertilization were used.

March 2021

Wild animals	no wild animals were used.
Reporting on sex	Sex-determination is not possible in the aforementioned age period.
Field-collected samples	no field-collected samples were used.
Ethics oversight	Secretariat of the Scientific Ethics Council for Animal Experiments (SSCA)

Note that full information on the approval of the study protocol must also be provided in the manuscript.
